# Ergo4Workers: A User-Centred App for Tracking Posture and Workload in Healthcare Professionals

**DOI:** 10.3390/s25185854

**Published:** 2025-09-19

**Authors:** Inês Sabino, Maria do Carmo Fernandes, Ana Antunes, António Monteny, Bruno Mendes, Carlos Caldeira, Isabel Guimarães, Nidia Grazina, Phillip Probst, Cátia Cepeda, Cláudia Quaresma, Hugo Gamboa, Isabel L. Nunes, Ana Teresa Gabriel

**Affiliations:** 1 Research and Development Unit for Mechanical and Industrial Engineering (UNIDEMI), Department of Mechanical and Industrial Engineering, NOVA School of Science and Technology, Universidade NOVA de Lisboa, 2829-516 Caparica, Portugal; 2 Área de Medicina Física e Reabilitação, Hospital Curry Cabral, Centro Hospitalar Lisboa Central, 1069-166 Lisboa, Portugal; 3 Laboratory of Instrumentation, Biomedical Engineering and Radiation Physics (LIBPhys-UNL), NOVA School of Science and Technology, NOVA University of Lisbon, 2829-516 Caparica, Portugal; 4 Associated Laboratory in Translation and Innovation Towards Global Health (REAL), 2829-516 Caparica, Portugal; 5 3D Printing Center for Health, Campus da Caparica, 2829-516 Caparica, Portugal; 6 Laboratório Associado de Sistemas Inteligentes (LASI), 4800-058 Guimarães, Portugal

**Keywords:** work-related musculoskeletal disorders, wearable sensors, smartphone application, usability

## Abstract

**Highlights:**

**What are the main findings?**

- The E4W system is a smartphone app that integrates data from wearable sensors.
- Three prototypes were developed following the UCD approach.
- This paper describes the development and usability evaluation of the E4W app.

**What is the implication of the main finding?**

- It provides insights about posture and workload for occupational therapists.
- Results evidence an easy-to-use and intuitive smartphone app.

**Abstract:**

Healthcare professionals (namely, occupational therapists) face ergonomic risk factors that may lead to work-related musculoskeletal disorders (WRMSD). Ergonomic assessments are crucial to mitigate this occupational issue. Wearable devices are a potential solution for such assessments, providing continuous measurement of biomechanical and physiological parameters. Ergo4workers (E4W) is a mobile application designed to integrate data from independent wearable sensors—motion capture system, surface electromyography, force platform, and smartwatch—to provide an overview of the posture and workload of occupational therapists. It can help identify poor work practices and raise awareness about ergonomic risk factors. This paper describes the development of E4W by following a User-Centred Design (UCD) approach. The initial stage focused on specifying the context of use in collaboration with six occupational therapists. Then the app was implemented using WordPress. Three iterations of the UCD cycle were performed. The usability test of prototype 1 was carried out in a laboratory environment, while the others were tested in a real healthcare work environment. The Cognitive Walkthrough was applied in the usability tests of prototypes 1 and 2. The System Usability Scale evaluated prototype 3. Results evidenced positive feedback, reflecting an easy-to-use and intuitive smartphone app that does not interfere with daily work activities.

## 1. Introduction

### 1.1. Background and Problem Description

The healthcare sector is one of the high-risk sectors regarding the prevalence of work-related musculoskeletal disorders (WRMSD) [1,2]. In a recent literature review of 18 studies on this topic, the authors analysed healthcare professionals’ experience with WRMSD, which often results in pain in the lower back, neck, arms, and knees [3]. Furthermore, a study carried out in 2018 in a Portuguese hospital with a sample of 105 healthcare professionals reported that the physical features of this job lead to exposure to various risk factors [1]. More specifically, activities related to the mobilisation of patients, such as carrying, transferring, or relocating patients, are pointed out as the main contributors to these exposures [4].

The term WRMSD refers to musculoskeletal disorders (MSD) that are significantly caused or exacerbated by occupational risk factors [5]. Although psychosocial and individual risk factors have been found to account for the prevalence of these disorders, these cannot, on their own, contribute to their development [6,7]. Physical risk factors for WRMSD include posture, force, and repetition [8].

A synthesis report published in 2020 by the European Agency for Safety and Health at Work, consolidating data from ten national reports across Europe, highlights the substantial impact of WRMSDs on a significant percentage of workers. The report also outlines the economic and social costs associated with the prevalence of WRMSD, which are anticipated to increase in the coming decades due to the ageing of the working population [9].

Therefore, implementing concrete strategies to deal effectively with WRMSD is crucial. These should include the performance of regular and thorough ergonomic assessments [5,8], which focus on identifying existing or potential occupational risk factors. There are three categories of risk assessment methods: self-reports (mainly through questionnaires), observation methods (which rely on an external observer), and direct measurement methods [10]. In a study focused between 2005 and 2018, the authors noted that certified ergonomic practitioners were increasingly using observational techniques [11], such as the Rapid Upper Limb Assessment (RULA) [12] and the Rapid Entire Body Assessment (REBA) [13].

Nonetheless, the subjective nature of these methods is linked to lower accuracy and precision compared to techniques that capture objective data [10,14]. Objective data can be assessed through direct instrumental measurements conducted by mechanical or electronic devices directly attached to the subject, enabling the measurement of biomechanical and physiological parameters [15].

### 1.2. Application of Wearable Technology and Related Work

The technological evolution associated with the emergence of Industry 4.0 has brought the integration of wearable sensors to the performance of ergonomic risk assessments [16,17,18,19]. These devices offer the possibility of performing a reliable and non-invasive assessment of the risks to which workers are exposed [14,19]. Furthermore, wearable technology continuously monitors human performance, even in complex occupational environments [20].

Due to its many advantages, the application of wearable technology has shown great potential for the improvement of the health, productivity, and safety of workers in a wide range of applications [14,17,19,21]. Wearable devices applied in ergonomic risk assessments include inertial measurement units (IMUs), motion capture (MoCap) systems, surface electromyography (sEMG) sensors, and insoles, which measure reaction forces [15,19].

There has been a growing interest in developing interactive systems using wearable sensors to assess workers’ exposure to occupational risk factors. These systems are designed to capture and analyse data in real time, providing valuable information as an output. Information about joint angles, body orientation, and movement is essential for applying ergonomic assessment methods, such as RULA and REBA. Traditionally, the necessary information was obtained by observation and video recording. Wearable technology allows for more data to be collected quickly and accurately [16,22,23].

Motion Capture Systems use different technologies, including optical (marker-based or markerless) and sensor-based IMUs, to capture posture and movement data. Optical MoCap, which relies on cameras and reflective markers to track movement, is widely recognised for its precision. However, they can only be used in controlled laboratory environments due to their need for calibrated camera setups, controlled lighting, and unobstructed line-of-sight between markers and sensors. Markerless MoCap systems remove the need for markers but may suffer from lower accuracy in complex work environments. Sensor-based IMUs, on the other hand, are portable and capable of capturing detailed kinematic data in real-time, making them particularly useful for ergonomic assessments in dynamic settings. IMUs and MoCap systems are particularly valuable for capturing kinematic data such as joint angles, posture, and movement, which are critical for assessing ergonomic risks [24].

Surface Electromyography sensors are used to measure electrical activity in muscles, which allows for the evaluation of muscle engagement and Maximum Voluntary Contraction (MVC). High muscle activation levels correlate with an increased risk of strain and musculoskeletal disorders [25].

Several studies have been conducted in this field, highlighting notable contributions. Firstly, in 2021, Zhao and colleagues presented a system based on IMUs that provides real-time and periodic (e.g., every 30 min) risk assessments of postures adopted by construction workers. The researchers developed their own mobile application prototype to communicate these results to end-users. They also conducted evaluations involving construction workers and managers [26]. Secondly, two studies published in 2020 describe the design and implementation of graphical user interfaces (GUIs) that provide visual feedback on the level of exposure to risk factors in the workplace [18,27]. These interfaces offer intuitive representations of the data collected by wearable sensors, enabling users to monitor and interpret risk levels effectively. Lastly, Vega-Barbas and his colleagues proposed and validated a comprehensive platform to prevent WRMSD by integrating wearable technology and a mobile app. The platform was tailored for two distinct occupational environments, ensuring applicability across different work settings [28]. These advancements demonstrate the ongoing efforts to leverage wearable sensor technology and interactive systems to improve the assessment and prevention of occupational risks. Integrating real-time data capture, analysis, and user-friendly interfaces holds great potential for enhancing workplace safety and promoting workers’ well-being.

The feedback can also be provided to end-users in real-time using systems whose main aim is to reduce and prevent exposure to WRMSD by directly monitoring subjects in their workplace [29,30]. Furthermore, examples of haptic feedback include sensor-based systems that warn workers in real time about postures and movements, which expose them to risk for WRMSD [31,32].

Most studies employ Artificial Intelligence algorithms to analyse measurement data and estimate ergonomic indexes, a subject extensively reviewed by Donisi and colleagues [17].

Furthermore, ERGO_X and FASTO ERGO_X are dedicated ergonomic analysis tools designed to identify, analyse, and mitigate potential ergonomic risk factors in occupational settings. These systems integrate concepts from fuzzy theory and artificial intelligence methods, facilitating users in systematically assessing relevant data. After this process, they recommend corrective measures to eliminate or reduce the identified risk factors [33].

In a parallel investigation, Halim and co-authors present the development of a user interface platform that integrates a postural assessment system with EMG. Notably, the authors explicitly indicate the absence of usability tests [34].

### 1.3. User-Centred Design Approach

Users of systems engage with equipment through interfaces, and this interaction is conditioned by their sensory, cognitive, and biomechanical capacities [35]. In contemporary times, numerous workers are obligated to interact with computer-based Information Systems, which can adversely affect them due to the cognitive demands they are exposed to [33].

User-centred design (UCD) is a methodological approach that strives to create usable, valuable, and user-friendly systems by actively involving users throughout the iterative design process and prioritising their needs and requirements [36]. The ISO 9241-210 standard [37], which focuses on human-centred design for interactive systems, delineates four fundamental activities applicable at any software development stage. These include understanding and specifying the context of use, considering the characteristics of the users, the tasks, and the organisational, technical, and physical environment in which the system is used. The succeeding activities are carried out iteratively to improve the product’s quality continuously [33,37] and include:

- Specify user and design requirements—this activity requires the identification of user needs and the characterisation of the functional requirements of the system;
- Solution design and implementation—this activity is fundamental to producing design solutions (e.g., mock-ups and prototypes) that meet the requirements;
- Evaluation—Even in the early stages of product development, design solutions should be evaluated against requirements to determine whether users’ needs are met.

Designing an interactive system—such as mobile apps—using a UCD process can improve users’ quality of life since the application of usability techniques and knowledge potentiate more intuitive, efficient, memorable, effective, and pleasant Human-system interaction. Furthermore, during the development cycle of a mobile app, it is fundamental to assess its accessibility. A system’s accessibility is related to the existing barriers between the system and user communication, and it focuses on guaranteeing the digital inclusion of users [33].

Usability is a feature of interaction between the user and the system and depends on the effectiveness, efficiency, and satisfaction with which users can perform a specific set of tasks in each context of use [33,38]. This concept is associated with the user experience (UX), which includes the perceptions and responses expressed by an individual during the interaction with any interface [37]. Thus, the usability of a system can be determined by assessing the accuracy with which users achieve a given goal when interacting with the system (effectiveness), the number of resources required to do so (efficiency), and their emotions during this interaction [39].

Testing for usability is a crucial component to include in the development process of user-centred systems [40]. Several usability methods and tools have been developed for a UCD process [33]. Deciding on the method to apply for successful results implies determining what information is needed, at what stage of system development the assessment takes place, and the resources available [33,40].

### 1.4. Aim of the Study

This study aimed to develop the Ergo4workers (E4W) designed for use in the occupational therapists’ work environment. The app was developed based on a UCD approach, and it integrates biomechanical and physiological data collected with wearable systems. These wearables include a motion capture system, a force platform, a surface electromyography system, and a smartwatch, each operating independently. Each wearable system already provides information, but E4W consolidates this data into a single platform, providing a centralised location for all the collected information to make it easier for users to access and interpret results. The gathered data can be relevant for identifying physical workload and gaining an ergonomic analysis.

The app is intended for periodic assessments (nevertheless, daily acquisitions are possible). Overall, the goal is to allow periodic individual monitoring of occupational risk factors, self-identifying potential ergonomic risks, and raising awareness of the necessity of adjustments to reduce the likelihood of developing WRMSD.

The main objectives of this paper can be defined as follows:

- Integrate biomechanical and physiological parameters from various sensor systems into one app, streamlining the collection and analysis of data in a single, centralised location.
- Develop a functional app tailored for occupational therapists.
- Apply the UCD approach throughout the app development process
- Provide reports at the end of each data acquisition session, summarising the key findings about posture and muscular effort (with the clearest graphics provided by each one of the sensors).

Six occupational therapists from a Portuguese Hospital participated in the study to achieve the goals. The volunteers cooperated to identify the users’ needs and the app’s requirements. The app’s design was inspired by PrevOccupAI, an app proposed for assessing exposure to risk factors in an occupational setting [41].

In line with the UCD approach, three prototypes were developed and tested for usability. The final prototype achieved a favourable score on the System Usability Scale questionnaire. It points out that the participants did not find significant usability problems when interacting with the app, and they considered that the information provided could help them improve their work practices.

## 2. Methodology

**Figure 1:**
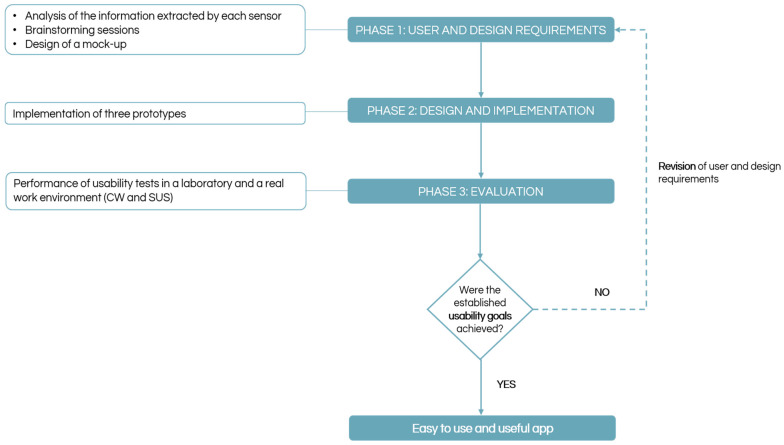
Methodology carried out in the development of E4W.

Figure 1 shows the design methodology used in this study. From the outset, a co-creation methodology was adopted, ensuring active participation of occupational therapists throughout the development process. This approach involved collaborative sessions to identify user and functional requirements with feedback on an initial visual mockup designed before any functional prototyping.

The methodology followed the three phases foreseen by the UCD approach (specifying user and design requirements, solution design and implementation, and evaluation). A subset of potential users, comprising six occupational therapists from a Portuguese hospital, actively participated in this process.

Phase 1, carried out after understanding and specifying the context of use, was initiated by identifying the equipment needed and understanding the information provided by each sensor. Then, it allowed the design of a mock-up resulting from a brainstorming session between the research team members. This design solution was then presented to the subset group of potential users, and a brainstorming session with the therapists was performed. Through this exchange of ideas, the needs of the occupational therapists were identified, and the app requirements were specified. The following phases—phases 2 and 3—consisted of the implementation and consecutive evaluation of the design solution (i.e., a prototype). The evaluation of each prototype was based on the application of usability evaluation methods, which made it possible to ascertain whether the prototype developed corresponded to the usability objectives outlined for the app. In each iteration, the research team analysed and discussed the results obtained, and improvement proposals were identified to be implemented in the following iteration.

Three iterations of the E4W mobile app development cycle were performed, and a final prototype (prototype 3), which represented an easy-to-use and helpful app, was implemented according to the results obtained.

### 2.1. Phase 1: User and Functional Requirements

For the specification of E4W’s user and design requirements, it was essential to identify the users’ needs and, firstly, to understand and specify the context of use (e.g., the occupational therapists’ workplace). Thus, an observation of the morning and afternoon shifts of the service was performed. The following topics were considered for this observation: the therapist’s name, the type of disorder treated, and duration of each treatment, a description of the adopted postures and body segments used more frequently, and relevant characteristics of the physical work environment, such as the workplace layout.

Afterwards, the authors found that creating a mock-up as a simple design suggestion would be beneficial, enhancing the main functionalities proposed for the app. Therefore, the first stage of this design process consisted of understanding the information extracted by the sensors, a topic that is further developed in the following subsection. The brainstorming technique was then applied by involving the team of occupational therapists, and its output was a collection of their objectives, needs, and preferences. Brainstorming promotes the exchange of creative ideas by allowing every participant to share and accept the design ideas produced [33]. As a result, the functional requirements of E4W were identified. These requirements included specifications related to the app’s content and design that should be verified to satisfy the users’ needs.

### 2.2. Phases 2 and 3: Implementation and Evaluation of the Design Solution

After specifying the requirements, the app’s three prototypes were implemented and evaluated. These prototypes evolved through an iterative design and evaluation process. In this cycle, the prototype is assessed according to its level of correspondence with user needs, and improvement proposals are identified. A new and improved prototype is developed by implementing improvement opportunities in an iteration cycle. Three iterations were performed throughout the development cycle of E4W since prototypes 1 and 2 were modified according to the results obtained in the usability tests.

The prototypes’ usability evaluation was carried out through usability testing. Informed consent was sought from each participant at each testing phase. Tests’ execution followed a script. A pilot test was conducted prior to the performance of the usability tests on prototype 1 with a participant familiar with the technology employed in E4W to validate the script and the analysis procedures. Thus, pilot testing leads to more reliable results in usability studies by identifying problems regarding the wording of the tasks or by helping to establish the time required for each task scenario [42].

This evaluation focused on measuring two main aspects of UX: performance and satisfaction. Usability metrics of task success, the time required to perform each task, the number of errors, actions performed, and thoughts or suggestions expressed were collected by applying two different usability evaluation methods: Cognitive Walkthrough (CW) and System Usability Scale (SUS).

CW was applied in two initial iterations of the app’s development cycle. Its main goal is to assess how users explore and become familiar with systems by analysing their mental processes while executing a task without prior training [33,37,43]. Three task scenarios were selected based on these two evaluation moments’ most essential and representative tasks. The description of the scenarios was written on a sheet of paper and placed next to the participant. During the test, interaction with the participant was avoided. After completing the tasks, participants were asked to comment on their interaction with the interface and to suggest improvements to be implemented, following a process described in a previous study [44].

Seven volunteers evaluated prototype 1 in a laboratory setting. As the purpose defined for this evaluation was to eliminate the most evident usability problems, namely regarding the general functionalities of the proposed design solution, it was deemed that these participants gathered the individual characteristics required to fulfil this goal. Furthermore, the disruption of occupational therapists’ work was avoided. After the redesign, one of the occupational therapists performed the usability testing of prototype 2 in a real work environment. This evaluation aimed to determine whether the modifications implemented in prototype 1 were adjusted to the needs of the group of potential users and if any more usability problems need to be addressed.

At a more advanced stage of the app’s development, SUS was applied to evaluate the usability of the final prototype. It consists of a usability questionnaire that provides a global vision of the user’s subjective impressions regarding the interaction with an interface [36,45,46]. This questionnaire comprises ten items that alternate in their connotation (1, 3, 7, and 9 are worded positively, and 2, 4, 6, 8, and 10 are worded negatively). These are scored using a 5-point Likert Scale, in which the users classify their level of agreement with each item. The five available responses range from Strongly Disagree to Strongly Agree [45]. The ten ratings are converted into an overall score on a scale from 0 to 100, and higher scores indicate better usability [39]. A validated European Portuguese version of the questionnaire was used [47]. Four occupational therapists were recruited to interact with the app and answer the questionnaire. Additional questions regarding the app’s functionalities were answered using the same 5-point Likert scale.

## 3. E4W App Architecture and Implementation

**Figure 2:**
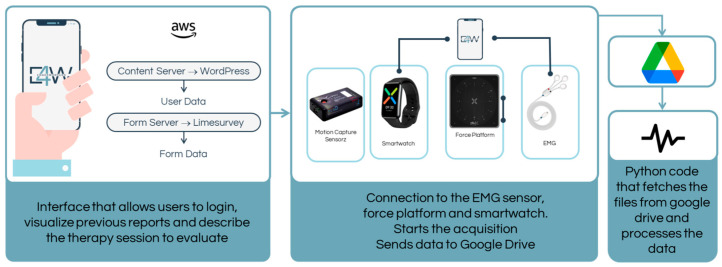
Architecture of the E4W application.

The app architecture is depicted in Figure 2. The application was deployed on an AWS virtual machine, using WordPress as the content server and LimeSurvey as the form server. WordPress, an open-source platform designed for creating blogs, websites, and applications, allows for the organisation of the application through widgets or in PHP and HTML code blocks. This platform streamlines content creation, enabling individuals without programming knowledge to utilise it efficiently while offering faster development capabilities for those with programming expertise. LimeSurvey, another open-source platform, is employed to create and develop online forms and to access form results and statistics without requiring extensive coding or scripting. The application was crafted within the WordPress framework, ensuring accessibility via both computer and mobile phone. After data collection, users can conveniently peruse reports for each data collection.

The Android mobile application establishes a connection with the web server via a JavaScript-designed interface. On the mobile phone, wearable sensors link to the application via Bluetooth, facilitating the data acquisition session. The acquired data is stored locally on the mobile phone as a .txt file and is concurrently uploaded to a designated Google Drive folder. Python code (version 3.10) is then employed to analyse the data, generating a conclusive report that is subsequently uploaded within the application.

It is noteworthy that the E4W app relies on outputs generated by the proprietary software of each commercial sensor system, which provides processed, validated data. These outputs are integrated into the E4W interface to consolidate relevant information from multiple validated sources into a single, intuitive platform.

### 3.1. Equipment

The equipment for real-time acquisition was selected based on the necessary parameters to collect (according to the type of occupational therapists’ work and the primary needs identified by the users) and existing equipment among the research team. It comprises a motion capture system, a smartwatch, an electromyography system, and a force platform. This set of devices works separately. Each incorporates many sensors to collect relevant parameters that help understand the physical workload. Each selected sensor plays a crucial role in comprehensively assessing ergonomic risk factors. By combining data from IMUs, sEMG, force platform, and a smartwatch, the E4W app can provide a holistic view of the occupational therapist’s physical workload.

**Figure 3:**
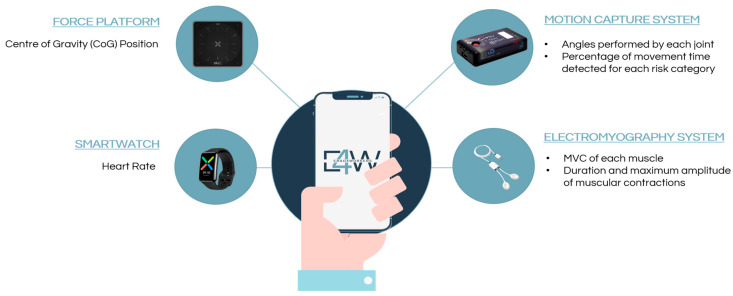
Representation of the parameters included in E4W (The image of each sensor was taken directly from the supplier’s website: Motion Capture System—www.teaergo.com/captiv/, Electromyography System and Force Platform—www.pluxbiosignals.com/, Smartwatch—www.oppo.com/pt/accessories/watch/, accessed on 15 May 2025).

Among the parameters collected with the wearable sensors, the ones defined as more relevant were aggregated and presented in the interface. Figure 3 identifies all the parameters acquired from the devices, such as the exerted force, body posture, angles associated with the movements, and centre of gravity (CoG) position.

#### 3.1.1. Motion Capture System

The motion capture system used to evaluate the subject’s posture and movement is an ergonomic system developed by the TEA company. This system, known as TEA Captiv T-sens Motion (IMU), comprises wireless inertial sensors with integrated magnetometers, accelerometers, and gyroscopes for motion detection. The sensors are fixed with straps and placed on the subject.

The parameters extracted from this motion capture system are the angles performed on each joint and the percentage of movement time detected for each risk category. These angles’ measurements result from the interaction between two sensors placed before and after a joint. CAPTIV has software that calibrates the sensors, allows the start and end of acquisition sessions, and collects information about them. The sampling rate is 64 Hz. After an acquisition session, the software processes the signal and estimates a risk evaluation in a colour code. The green, orange and red colours are assigned according to thresholds for the joint angles predefined by the CAPTIV software. The system’s risk assessment is based on a validated model [48]. The CAPTIV results can be extracted into a PDF or HTML file.

#### 3.1.2. Surface Electromyography System

The sEMG system chosen to monitor and evaluate the muscle activity performed by healthcare professionals was the EMG equipment (developed by a company named Plux Biosignals S.A.), consisting of an 8-channel hub that connects up to eight electromyography sensors (Information obtained from the website www.pluxbiosignals.com/).

Each electromyography sensor has a bipolar configuration that must be placed on the muscle group to be evaluated, with a distance of two centimetres between the two electrodes. This equipment acquires electrical muscle activity with a sampling frequency of 1000 Hz and a resolution of 16 bits. The data is transmitted via Bluetooth to the device, where the acquisition is performed, and compressed into a folder sent to Google Drive.

The collected data were processed in Python and normalised to determine the specific muscle’s maximum voluntary contraction. The MVC is the maximum force the individual exerts voluntarily, allowing for the calibration of the sEMG signal. At the start of an acquisition session, the healthcare professional was asked to perform the maximum voluntary contraction on the muscle to be evaluated through the performance of specific exercises. This contraction was repeated three times, and the contraction with the highest value was considered the MVC. The data acquired is assessed according to the percentage of MVC, where four different colours can be assigned according to the risk degree associated with the force intensity: green corresponds to low intensity (0%MVC–20%MVC), yellow is attributed when a moderate level of intensity is applied (21–40%), orange is associated with a high level of intensity (41–60%), and an MVC of greater than 60% corresponds to the colour red. The thresholds were used to alert the user to the muscular effort during an acquisition [49].

#### 3.1.3. Force Platform

Plux developed the selected force platform, which comprises four load cells positioned at each platform corner. These load cells are connected to a hub, enabling data transmission via Bluetooth between the hub and the acquisition device. The data transmission occurs at a frequency of 1000 Hz and has a resolution of 16 bits.

The force platform allows for the extraction of essential parameters related to force, including the maximum force exerted within a defined time interval and parameters associated with jumps, such as jump duration. In this study, the Force Platform was utilised to extract the CoG, and the corresponding results can be found in the final report accessible through the application.

#### 3.1.4. Smartwatch

The E4W app also uses the data collected by a smartwatch’s heartbeat sensor to acquire the healthcare professional’s heart rate during the data collection sessions. It transferred the data acquired using an Oppo smartwatch, which is supported by Android devices.

### 3.2. Equipment Configuration

**Figure 4:**
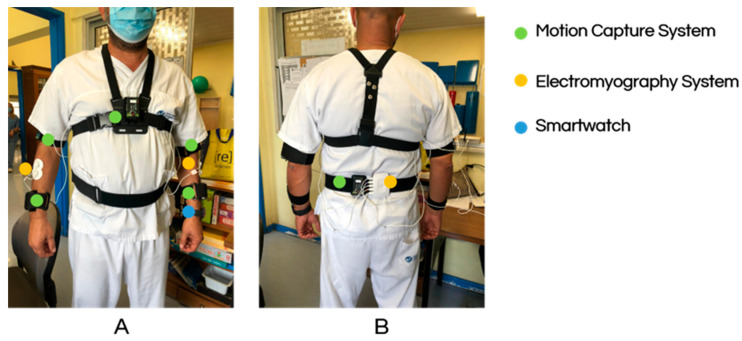
Demonstration of the sensors’ placement on an occupational therapist (**A**) Anterior side of an occupational therapist with the motion capture sensors and the electromyography system; (**B**) Posterior side of the occupational therapist with both motion capture and surface electromyography sensors.

Figure 4 shows an occupational therapist wearing different devices for the acquisition session. The marked bands correspond to the motion capture sensors, the white electrodes are the EMG system, and the smartwatch is displayed.

Since these professionals were observed to perform their work activities mainly seated and according to identified ergonomic evaluation needs, the upper limbs were selected to be monitored. Eight electrodes were used to collect sEMG signals from two muscles in each upper limb: the deltoid and extensor pollicis muscles. Although it can also be used in the standing position, the force platform was used on a chair where the therapists sat for the evaluation, given the position they mainly adopted. Determining the CoG enables a more complete evaluation of the posture adopted when combined with the motion capture system.

A series of acquisition sessions involving four distinct occupational therapists was carried out. Given the app’s early development stage, the research team was responsible for sensor placement and calibration. No issues were identified in capturing the intended parameters throughout the acquisition sessions, and the participating therapists did not report any discomfort or disruption while performing their tasks.

### 3.3. Assessment Report

Although the devices work independently, the E4W app integrates the data collected from each sensor to provide a comprehensive report. Each wearable provides specific insights into different aspects of the therapist’s physical workload, such as posture, muscle strain, force exertion, and cardiovascular strain.

The report becomes available at the end of each acquisition and can be accessed anytime by the user. The information is directly extracted from the software of each wearable device. The motion capture system provides an avatar that represents the worker’s activity, and the monitored joints have a colour (green, yellow or red) to identify the low, medium and high risk of developing WRMSD due to posture [50]. The sEMG software creates a three-bar chart with a similar colour code that gives an idea of the time spent without exerting force, exerting a medium force, and exerting a high force (in terms of %MVC). The data extracted from the force platform is a plot that relates the CoG to time. Finally, the heart rate measured by the smartwatch is plotted as a function of time.

## 4. Results and Discussion

This section describes in detail and discusses the results obtained from the iterative process employed in developing E4W’s app, which resulted in three iterations. Aligned with the principles of the UCD approach, the development of E4W involved a co-creation process with six occupational therapists from a Portuguese hospital. These professionals actively contributed to all stages of development.

Firstly, in phase 1, the user and design requirements, defined according to the specification of the context of the use of E4W and the needs of occupational therapists, are detailed. Afterwards, the three prototypes implemented are described in detail (Phase 2), and the main results obtained for their usability evaluation, performed in Phase 3 of the methodology, are reported.

### 4.1. Phase 1: Specifying User and Design Requirements

As previously mentioned, E4W’s end users are, in the current research, healthcare professionals. This paper describes a study focused on a subgroup of potential users: occupational therapists. Thus, the app’s design solutions were developed and designed by assessing the needs of a team of six occupational therapists working at a central Portuguese hospital. Table 1 summarises the app’s functional requirements according to the team of occupational therapists’ assessment of their needs.

E4W is intended to provide an integrated overview of posture and workload of occupational therapists’ work activities by allowing them to perform acquisitions in which data is extracted from four different sensor systems: a motion capture system, a surface electromyography system, a force platform, and a smartwatch. An acquisition session corresponds to a therapy session, which lasts an average of 30 min. According to the patient’s condition, the physical condition of the work performed by the therapists (e.g., if they are standing or sitting, the posture adopted, the body extremities and muscles used more demanding) varies. Also, their workspace has different features: the session can take place in two different rooms, and, therefore, the healthcare professional can be seated in chairs with different characteristics and at tables that differ in height. These aspects of the therapy session influence the occupational therapist’s risk exposure.

Thus, the E4W app allows assessing reports that display information about the posture and workload of each therapy session based on the data acquired by the sensor systems. E4W’s input also includes relevant characteristics of each session, which the users register through the submission of a survey. By introducing these characteristics, users know the conditions under which the session was held, which can be relevant to identifying the work conditions associated with a higher risk.

### 4.2. Phases 2 and 3: Implementation and Evaluation of the Design Solution

Three prototypes with different degrees of realism and sophistication were designed for E4W’s app. The following sections present each prototype and the main results obtained for its usability evaluation.

#### 4.2.1. Prototype 1

The initial prototype was developed based on the previously described functional requirements.

The user begins logging in using their email and password during the app’s interaction. Upon successful login, the user is directed to the application’s home page, where two options are available: perform an acquisition session or access previous reports. The main menu also provides options to log out, obtain information about the E4W app, and return to the home page.

Initiating an acquisition session involves a three-step process presented to the user sequentially. First, it is crucial to properly position and calibrate the sensor systems that will be used to measure ergonomic evaluation parameters. One of E4W’s long-term objectives is to enable users to perform acquisition sessions independently. Therefore, providing explicit and informative content about the sensors within the app is essential. Subsequently, a survey designed to capture relevant aspects of a therapy session is presented to the user. The survey questions were formulated based on inputs from occupational therapists during brainstorming sessions and consideration of the usage context. Finally, by clicking the designated button, the data from the sensors will begin recording. To access and review the previous reports, the user can navigate to the “Registration of Assessments” section on the home page and select the desired report.

Since this is the initial design iteration, certain functionalities, such as the sensor information section, are not yet fully developed.

This prototype was evaluated through usability tests performed in a laboratory environment with seven participants using the CW method. Performance and satisfaction metrics were collected and analysed. Data regarding performance metrics included the task’s success, the time required to perform each task, the number of errors, and the actions performed. At the end of the test, participants’ feedback was collected about their interaction with the interface. Overall, the results evidenced a positive evaluation of the prototype since approximately 90% of the tasks were completed with few errors. Also, favourable feedback was obtained, as participants mentioned the app’s intuitive functionalities, usefulness, and appealing features. However, some usability problems were experienced, namely regarding the app’s layout and the consistency of the wording used, and improvement proposals were made to be addressed in the following iteration so that the UX was improved [51].

#### 4.2.2. Prototype 2

Based on the usability problems identified in the usability evaluation of prototype 1, modifications were implemented on the app’s second prototype (Table 2).

These improvements focused on modifying the page providing information on the sensors. Participants of the usability tests mentioned that the wording used was unclear regarding the following action to perform, and that specific layout aspects affected their interaction with the prototype. Similar modifications were implemented on the survey to characterise the therapy session and the app’s homepage.

A usability test, performed by an occupational therapist, was carried out to evaluate prototype 2 in the participants’ workplace. This evaluation occurred under the same conditions as the first iteration, including applying the CW method. Since the main focus of the evaluation consisted of assessing whether the modifications implemented in prototype 2 met the user requirements, it was considered that one participant among the sample of six occupational therapists would be sufficient to achieve this goal. The results were compared to those recorded from the previous evaluation, and they revealed that the number of usability problems identified in prototype 2 significantly decreased compared to prototype 1. Throughout the test, the participant’s satisfaction and positive feedback were consistent with the performance metrics results, namely the 100% task success without errors [52]. Thus, it was possible to conclude that prototype 3 could be implemented as a more detailed design solution for the E4W’s app, given that this prototype—consisting of a simpler version of the app—matched the needs of this subset group of potential users.

#### 4.2.3. Prototype 3

The final prototype was created during the third and final iteration of the E4W development cycle. Following the usability test conducted in an occupational setting, a minor modification was made to the survey regarding the presentation order of answer options for a particular question.

**Figure 5:**
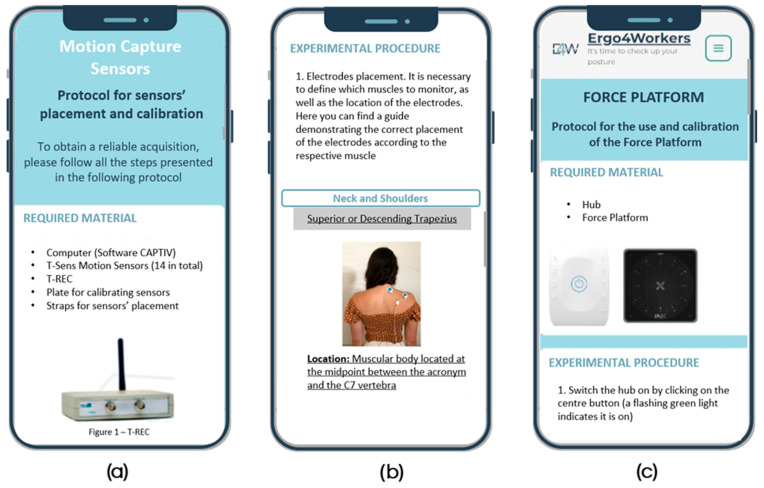
Examples of E4W’s final prototype interfaces on sensors’ placement and calibration. (**a**) Motion capture system; (**b**) Electromyography system; (**c**) Force platform.

Additionally, a functional requirement about including sensor placement and calibration information was addressed. Dedicated pages were added for each wearable sensor system to fulfil the previous requirement. These pages provided detailed descriptions of the necessary materials and step-by-step instructions for proper acquisition preparation (refer to Figure 5). Accompanying images were incorporated alongside the textual content to enhance user comprehension and facilitate the process.

**Figure 6:**
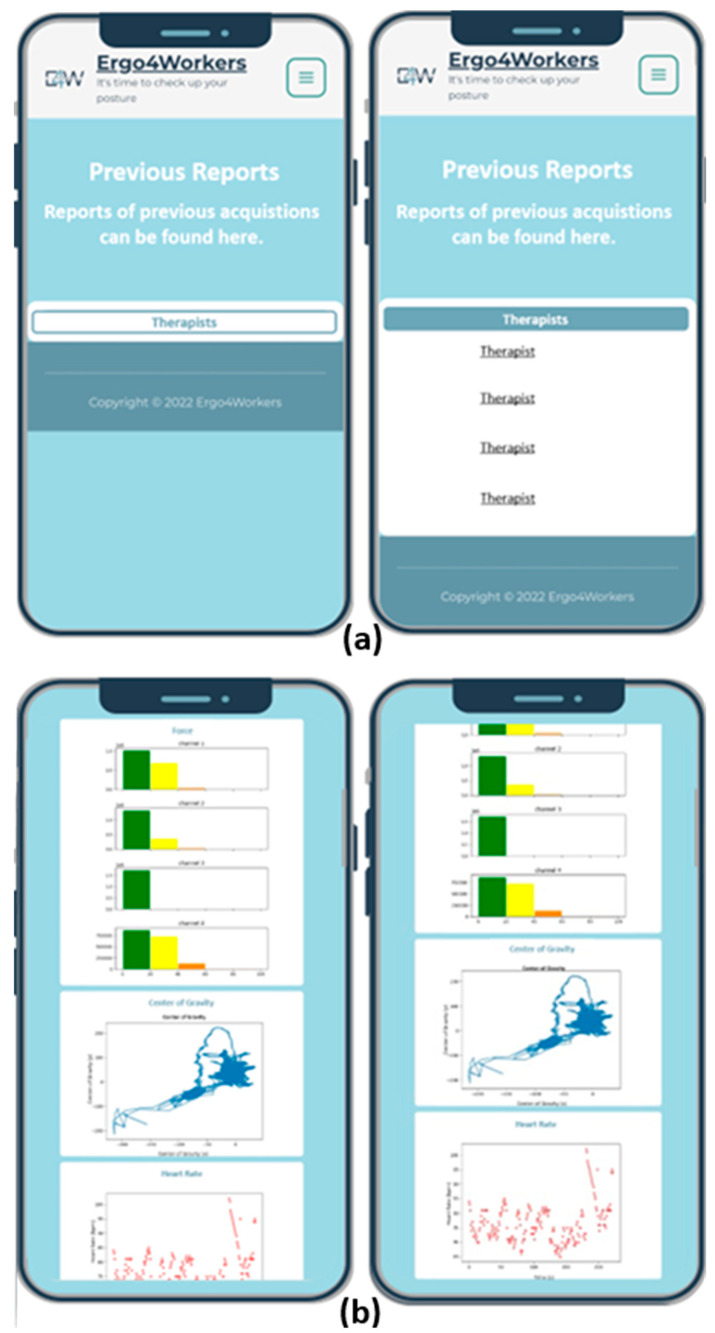
Examples of the interfaces of E4W’s final prototype regarding viewing previous reports. (**a**) Selecting the report; (**b**) Graphs designed according to the parameters acquired by each wearable equipment.

Figure 6 shows the implementation of the functional requirement regarding accessing previous reports in the final prototype. As previously mentioned, eleven acquisitions of four occupational therapists were carried out to develop this app’s functionality. By clicking on the button corresponding to each therapist, the user obtains the report regarding the information collected during the acquisition. For privacy reasons, occupational therapists’ names were omitted in Figure 6. Each report includes three graphs displaying the parameters acquired by the wearable equipment and the report provided by the software CAPTIV on the percentage of motion time detected for each risk category.

Regarding the usability evaluation of this prototype, a sample of four occupational therapists filled in the questionnaire SUS regarding the final prototype of E4W’s app. Table 3 presents the final SUS scores obtained from each participant and the respective mean score and 95% confidence interval. The confidence interval was calculated to demonstrate the variability associated with the individual scores attributed by each one of these four occupational therapists. A colour map was used to interpret these results, in which the final scores were converted into adjectives [53]. Thus, green is associated with scores between 80.8 and 84.0 and the adjective “Excellent”. The scores that classify the final prototype of the E4W app as “Ok” are highlighted in yellow, ranging from 51.7 to 71.0.

It is, then, possible to verify that participants P3 and P4 classified the prototype as “Excellent”, whereas the scores attributed by P3 and P4 resulted in a rating corresponding to the adjective “Ok”. Overall, these results indicate that the usability assessment was entirely satisfactory since the mean score indicates that the final E4W prototype was rated as “Good” by the therapists. However, both P1 and P2 assigned a final score below 68, interpreted as this app obtaining a usability evaluation that is below average compared to other products [47].

Table 4 presents the average values of the individual scores obtained for each item, allowing an analysis of these scores.

As shown in Table 4, among the positive items, item 7 was the only one with an average score below 4. Negative items 2, 4, 6, and 10 were rated with a score higher than 2. According to the participants’ comments, these results are mainly associated with the process required to start an acquisition. Participants P1 and P2 highlighted that they could not clearly understand this process, expressing the need to obtain training to achieve this goal initially. These observations point out that future developments of the application should focus on including video tutorials or a built-in step-by-step assistant to support users during session setup.

Furthermore, all therapists agreed with positive items 1 and 5. Thus, it is confirmed that this group of potential users positively assessed the most critical components of the prototype’s usability. Item 1, “I think I would like to use this product frequently,” was the statement with the highest score among the ten statements included in SUS.

Additional questions were placed for this usability evaluation. These were included to assess the prototype’s functionalities in greater detail, especially regarding their intuitiveness, simplicity, and clarity. Moreover, participants were questioned about functional requirements that were planned to be implemented in a future prototype. All participants demonstrated satisfaction regarding these features. These are the following:

- Provide information regarding each sensor’s placement and calibration process by viewing a brief explanatory video.
- Allows users to search for a previous report through filters defined according to the characteristics introduced for the therapy session.

Furthermore, the group of occupational therapists was also queried regarding their perception of the utility and interpretability of the graphs incorporated in the reports. Valuable insights and improvement opportunities were gleaned from engaging in discussions with these professionals and their perspectives and recommendations. During these conversations, three therapists highlighted the need for a more comprehensive description of the axes in the graph depicting the displacement of the CoG position, as measured by the force platform. In the final prototype, the axes are denoted as “x” and “y”. The suggestions were addressed to facilitate a more intuitive interpretation and included specifying the dimensions of displacement in terms of left, right, front, and back.

Compared to other existing tools that inspired its development, namely ERGO_X and PrevOccupAI, the E4W is distinguished by integrating multiple wearable sensors into a single platform, offering comprehensive biomechanical and physiological data to track posture and workload during practice. Additionally, E4W has been developed following a UCD approach tailored for occupational therapists, providing accessible and detailed reports. Table 5 summarises the key differences.

## 5. Conclusions

E4W was conceived to furnish healthcare professionals vulnerable to the onset of WRMSD with a streamlined and interactive platform that provides insights about posture and physical workload during routine tasks. The smartphone application integrates data from wearable sensor systems, reporting ergonomic factors associated with posture, muscle strain, and physical workload. The recent literature underscores the versatility of wearable devices across diverse occupational environments, demonstrating reliability and continuity in monitoring and assessing human performance. Therefore, E4W endeavours to empower healthcare professionals to acquire real-time biomechanical and physiological parameters, providing valuable insights on aspects such as posture within their working contexts. It aims to contribute to guiding their practice to prevent WRMSD.

The development methodology of E4W was anchored in a UCD approach, which proved instrumental in creating a user-friendly and efficient mobile application. Six occupational therapists were involved as active participants, and the iterative process persisted until predefined objectives were met. Functional requirements were established based on user needs, and prototypes evolved through iterative design and evaluation processes. Three prototypes were developed and assessed for usability using the CW and SUS, collecting pertinent metrics on usability and satisfaction.

The study represents proof of concept, focusing on usability and initial testing within a clinical context. The outcomes suggest that E4W has potential to positively impact on the healthcare sector. By leveraging innovative and non-invasive technology, the application offers relevant feedback to professionals. The UCD methodology is anticipated to reduce cognitive demand, ensuring the application’s ease of use and intuitiveness.

Despite the study’s contributions, several limitations should be acknowledged. One notable limitation stems from the small sample of occupational therapists available to collaborate with the co-creation process. For that reason, a laboratory-based usability test was performed involving participants, primarily students and faculty members. Although this group provided valuable feedback on the general usability and interface design, their lack of healthcare experience means that their feedback may not have fully captured the professional workflows and the assessments in healthcare settings. Students may also be less familiar with the practical demands in the healthcare sector, which could have led to differences in feedback compared to what would have been provided by actual healthcare professionals.

The previous limitation does not impact on the development of the E4W because two more interactions were performed. Also, the usability evaluation of the third and final prototype was satisfactory. Nevertheless, in future developments, simplifying the initiation of data collection should be prioritised. Feedback from two participants suggests that including short video instructions or a built-in step-by-step assistant could significantly improve the user experience, especially during the setup and calibration of sensor systems. These features may help reduce the learning curve and enhance the app’s accessibility for new users or those less familiar with digital tools.

Another limitation is the fact that the usability test of the final prototype involved a small number of occupational therapists, which may limit the generalizability of the findings to broader healthcare professional populations. It is necessary to acknowledge that healthcare professionals’ feedback is essential for understanding the specific requirements of the app, especially in terms of its usability, as they are the primary users. As such, it would be essential to involve a larger sample of healthcare professionals, ensuring that usability testing reflects the needs and working conditions of the app’s target users. It can provide a more accurate representation of how the app will function in real-world settings, leading to a more tailored and effective solution for tracking ergonomic risk factors in healthcare professionals.

A third limitation is that the application’s design was tailored to the specific needs and working context of a particular and small group of healthcare professionals (occupational therapists). Therefore, the functional requirements and preferences might differ if increasing the sample of occupational therapists or among different groups of healthcare professionals. Technological limitations were also found. The integration of the CAPTIV system posed challenges, particularly with the output format of evaluation reports, limiting the extraction of evaluation reports to .pdf format.

Future studies should address additional functional requirements identified by users and rectify usability issues. Extensive usability tests involving a bigger sample are necessary to refine and validate the mobile application further. In addition, to apply the E4W to other medical specialties it is essential to conduct a broader validation process involving professionals from different healthcare fields. This step is critical to confirm that the application meets the specific needs and working conditions of various user groups within the healthcare sector. The ultimate objective is to develop a user-friendly, sector-appropriate mobile application that is reliable, relevant, and straightforward for assessments in the healthcare workplace and that provides real-time feedback in addition to the final reports.

Despite the aforementioned limitations, the E4W app represents a first step toward advancing the assessment of ergonomic risk factors and contributing to the prevention of WRMSD among healthcare professionals. Unlike previous studies that relied on individual sensor technologies and were not specifically developed for the healthcare context, the E4W presents an integrated solution that combines four distinct wearable systems: inertial motion capture, surface electromyography, force platform, and smartwatch. This multi-sensor integration may offer a more comprehensive and multidimensional assessment of physical workload by capturing posture, muscle activation, balance, and activity patterns simultaneously. Moreover, the E4W distinguishes itself by combining this technical integration with a co-creation methodology and iterative UCD process further ensure that the tool is aligned with real-world needs and conditions in clinical practice.

In conclusion, results indicate that the E4W app demonstrates substantial promise as a user-friendly, data-driven tool for improving data collection within healthcare environments. With further refinement and broader implementation, E4W could contribute significantly to preventing and monitoring the risk of developing WRMSD among healthcare professionals, ultimately enhancing worker safety and productivity. E4W could also serve as a model for developing similar tools tailored to other healthcare settings, helping to strengthen the well-being of healthcare workers globally. Policymakers could support the integration of wearable technology in healthcare settings as part of a broader strategy for workplace safety and occupational health.

## Figures and Tables

**Table 1:** Functional requirements of the E4W app for the team of occupational therapists.

Functional Requirements
1. Allows users to log in and out of the account, enabling the differentiation of data from each user.
2. Allow data collection from different sensor systems
3. Provide clear and helpful information regarding each sensor’s placement and calibration processes
4. Allow registering, from a set of options, previously defined relevant characteristics of the therapy session (i.e., type of disorder of the patient, body regions in which they are being treated, and layout of the working space)
5. Allow the user to access individually their reports on previous assessments

**Table 2:** Modifications implemented for the design of prototype 2 of the E4W’s app.

Modification	Prototype 1	Prototype 2
Replacement of the button label “Start Measurement” with “Next.” Clarifying the page’s title and description Reduction of the button’s fields for each sensor	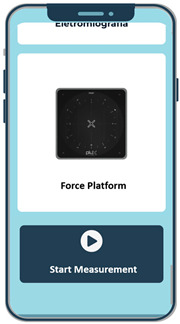	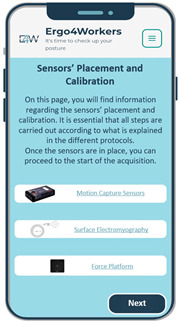
Addition of a direct and clear title Reduction of the field intended for filling in the questionnaire	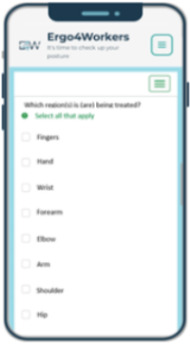	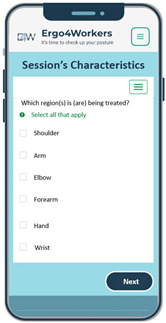
Replacement of the button label “Registration of Assessments” with “Consult Reports.”	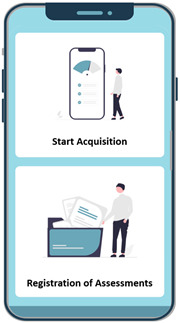	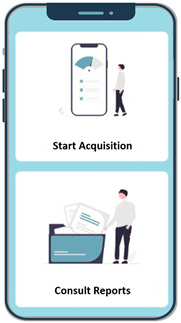

**Table 3:** SUS results.

Participant	SUS Score
P1	62.5
P2	65.0
P3	80.0
P4	82.5
Mean Score	72.5
Confidence Interval (95%)	12.0

**Table 4:** Average responses attributed to each SUS item.

Items	Mean SUS Score
1. I think that I would like to use this app frequently	4.5
2. I found the app unnecessarily complex	2.8
3. I thought the app was easy to use	4.0
4. I think that I would need the support of a technical person to be able to use this app	2.4
5. I found the various functions in this app were very well integrated	4.3
6. I thought there was too much inconsistency in this app	2.5
7. I would imagine that most people would learn to use this app very quickly	3.8
8. I found the app very cumbersome to use	1.5
9. I felt very confident using this app	4.0
10. I need to learn a lot of things before I can get going with this app	2.3

**Table 5:** Comparison between E4W and other existing solutions.

Characteristics	ERGO_X	PrevOccupAI	E4W
Target Population	General workers	General workers	Occupational therapists
Wearable sensors	None	IMU	IMU, sEMG, force platform, smartwatch
Primary objective	Psychological and postural risk	Upper limb WRMSD risk prediction	Track posture and workload
User Interface	Mobile app	Mobile app	Decision-support system (no dedicated app)

## Data Availability

The original contributions presented in this study are included in the article. Further inquiries can be directed to the corresponding author.

## Abbreviations

The following abbreviations are used in this manuscript:

CoG	Centre Of Gravity
CW	Cognitive Walkthrough
E4W	Ergo4Workers
EU-OSHA	European Agency For Safety And Health At Work
GUI	Graphical User Interface
IMU	Inertial Measurement Unit
MoCap	Motion Capture
MVC	Maximum Voluntary Contraction
REBA	Rapid Entire Body Assessment
RULA	Rapid Upper Limb Assessment
sEMG	Surface Electromiography
SUS	System Usability Scale
UCD	User-Centred Design
UX	User Experience
WRMSD	Work-Related Musculoskeletal Disorders
